# Spatial hotspot analysis of soil erosion rate and classification of homogeneous zones using GIS in a mountainous contrasting land-use watershed

**DOI:** 10.1038/s41598-026-41668-z

**Published:** 2026-02-25

**Authors:** Fatemeh Saeedi Nazarlu, Hassan Khavarian Nehzak, Raoof Mostafazadeh, Nazila Alaei

**Affiliations:** 1 Department of Remote Sensing, Faculty of Social Sciences, University of Mohaghegh Ardabili, Ardabil, Iran; 2 Department of Natural Resources, Faculty of Agriculture and Natural Resources, Member of Water Management Research Center, University of Mohaghegh Ardabili, Ardabil, 5951816687 Iran; 3 Department of Range and Watershed Management, Faculty of Natural Resources, Urmia University, Urmia, Iran

**Keywords:** Spatial data, Hotspot, Clustering, Moran’s index, Erosion modeling, RUSLE model, Climate sciences, Ecology, Ecology, Environmental sciences, Hydrology, Natural hazards

## Abstract

Soil erosion poses a significant challenge to environmental sustainability, especially in regions with varying land-use patterns and topography. Soil erosion is a major environmental threat affecting soil quality, reservoir sedimentation, agricultural land, and watershed hydrology. This study aims to identify and classify homogeneous sub-watersheds in a mountainous watershed in Iran using GIS. Forty years of climate data, a high-resolution DEM, land-use maps, soil texture, and NDVI were applied to derive the main factors, while the P factor was determined based on slope classes and land-use types. The RUSLE results showed that annual soil erosion in the watershed had an average of about 7-ton ha⁻¹ year⁻¹, with more than 65% of the watershed area falling into the moderate to very high erosion classes. Average key factors were *R* = 78.08 MJ·mm/ha·hr·year, K = 0.28 t·ha·h/MJ·mm·ha, LS = 1.62, and C = 0.39. The highest erosion occurred in areas with heavy rainfall, steep and long slopes, fine-textured soils, and sparse vegetation. Spatial autocorrelation analysis using Moran’s I and the Getis–Ord Gi* statistic showed a clustered spatial pattern of erosion. High–high (HH) clusters, indicating severe erosion hotspots, were found in the southwest, while low–low (LL) clusters, representing minimal erosion coldspots, occurred in the north and northeast. These results support sub-watershed prioritization and indicate the need for targeted erosion control in high-rate zones. These results contribute to the development of more targeted and sustainable land management practices to mitigate soil erosion rates and improve watershed conservation efforts.

## Introduction

### Background

Soil erosion is one of the major environmental challenges that leads to the degradation of soil quality, destruction of agricultural lands, water pollution, and sedimentation in reservoirs^1–3^. The loss of the fertile topsoil layer, increased surface runoff, sediment deposition in reservoirs, and reduced water storage capacity are just some of the consequences of this phenomenon^4^. Considering the direct and indirect impacts of erosion on agricultural production and sustainable development, identifying sensitive areas and implementing proper management in watersheds has become a vital necessity^2^. Advances in modern technologies such as remote sensing (RS), geographic information systems (GIS), and spatial statistics have enabled precise and regional analysis of soil erosion^5,6^. These tools can integrate various data, including topography, land use, precipitation, soil type, and vegetation cover, and can produce detailed erosion maps using models like RUSLE^7^. Spatial statistical analyses such as Moran’s Index also enable the identification of hot and coldspots of erosion, which are highly beneficial for conservation planning^8,9^.

Remote sensing (RS) and geographic information systems (GIS) are recognized as powerful tools for studying and modeling soil erosion. By using satellite images and remote sensing techniques, factors influencing erosion, such as vegetation cover, land slope, and land use, can be monitored with high accuracy^10^. On the other hand, GIS, with its spatial analysis capabilities and ability to integrate various data layers, allows researchers to efficiently apply erosion models like RUSLE (Revised Universal Soil Loss Equation) or SWAT (Soil and Water Assessment Tool). The combination of these two technologies not only enhances the accuracy of erosion estimates but also significantly aids in the optimal management of soil resources and conservation planning^11,12^. Recent research has shown that combining RUSLE with remote sensing data, especially the use of NDVI, improves the accuracy of the C factor calculation and facilitates the production of higher-resolution erosion maps^13^. Additionally, the use of precise elevation models, such as LiDAR-derived DEMs, has significantly improved the accuracy of the LS factor, and recent studies have shown that appropriate conservation measures can reduce erosion by 30 to 50%^14^.

### Literature review

Numerous studies have been carried out to assess the spatial variations of soil erosion in different regions of the world. In this context, Zhou and Wu^15^ in China used GIS and RS to evaluate the area of regions with varying erosion intensities and sediment delivery ratios in the Chaobaihe watershed. Bayat et al.^16^ in Lorestan Province integrated RS and GIS with the USLE model to prepare an erosion hazard zonation map. Their results showed that 54.5% of the area fell into the high-erosion class, with most critical zones located in the southern and southwestern parts of the province. Ahmadabadi and Sedighifar^17^ combined the RUSLE model with GIS and RS in the Hablehroud watershed, estimating an annual soil loss of 459 megajoules/mm/hectare/hour, indicating an urgent need for conservation practices. Ganasri and Ramesh^18^ used the RUSLE model and RS to evaluate soil erosion in India’s Nethravati watershed. Their results indicated that land-use changes directly contributed to increased erosion. Likewise, Tamene et al.^19^ in Ethiopia applied hotspot analysis to identify critical erosion areas, showing that prioritizing high-risk zones can help reduce sediment.

Alewell et al.^20^ used machine learning algorithms to develop a global soil erodibility map and found that intensively cultivated and deforested areas exhibited higher K values. In contrast, volcanic soils typically showed lower K values due to their high organic content. Madadi et al.^21^ in the Nirochay watershed found that among 25 geomorphic variables, four factors (area, form factor, perimeter, and discharge) had the greatest influence on sediment yield. Principal component analysis was used to identify the dominant factors.

Guo et al.^22^ in the Beijing–Tianjin–Hebei region conducted a multi-scale assessment and showed that despite an overall decline in erosion rates, some zones still experienced high-intensity erosion. Xo et al.^23^ used NDVI to analyze hotspots and spatio-temporal patterns in the Jingqin River watershed, revealing a declining vegetation trend strongly affected by rainfall and land-use changes. Jokar Sarhangi & Dehghan^24^ evaluated the performance of the RUSLE and ICONA models for erosion zonation in the Baladeh watershed of Mazandaran. Although neither model achieved high accuracy, the ICONA model showed better agreement with reference data. Hailu et al.^25^ in Ethiopia’s Tekeze watershed used sediment-yield modeling to identify six sub-watersheds as critical. Munye et al.^26^ through applying RUSLE and a multi-criteria decision analysis (MCDA) approach in Ethiopia, demonstrated that MCDA provided higher accuracy than traditional methods for identifying erosion-prone areas. Geospatial tools are now fundamental for soil erosion studies, with proven success in quantifying soil loss and locating critical areas through models like RUSLE integrated with GIS and remote sensing. The adoption of advanced spatial statistics, such as hotspot analysis using Moran’s I and Getis-Ord Gi* indices, has been a key advancement. These methods shift focus from general averages to identifying precise, statistically significant clusters of severe erosion, thereby generating actionable maps for targeted soil and natural resource management planning^27,28^. However, a significant research gap remains in applying these sophisticated techniques to mountainous watersheds with highly contrasting land-use patterns. Existing research often concentrates on areas with uniform land cover or emphasizes climatic and topographic drivers, while overlooking how the immediate adjacency of differing land uses (like intensive agriculture next to forests) shapes the formation and spatial structure of erosion hotspots in rugged terrains.

### Scope and objective

The Qara-Su watershed, located in Ardabil province, holds significant importance as a study area due to its diverse range of agricultural, industrial, and tourism activities. This region is also characterized by the presence of important population centers and protected areas, which have unfortunately been impacted by human activities^29^. The Qara-Su watershed in Ardabil Province, due to its distinct topographic characteristics, semi-humid climate, extensive agricultural activities, and sensitive land uses, is considered one of the erosion-prone regions in northwestern Iran^30^. Land-use changes, overgrazing, vegetation degradation, and the lack of sufficient field-based sediment data have exposed this watershed to severe degradation risks. The absence of adequate sediment-monitoring stations and long-term observational records further emphasizes the need for indirect and spatial analytical methods in this watershed.

This study aims to identify homogeneous sub-watersheds in terms of current soil erosion rates and sediment production potential in the Qara-Su watershed by employing the RUSLE model, spatial indices, and geospatial analytical tools. The core innovation of this research lies in its emphasis on spatial correlation analysis and the integration of remote sensing and GIS-derived datasets. Through mapping spatial patterns of soil erosion rates and analyzing hot and cold spots using Moran’s I and the Getis–Ord Gi* statistic, this study generates practical maps to support soil management planning and erosion-control strategies. The primary objective is to decipher how the specific spatial arrangement of contrasting land uses interacts with local topography to drive the clustering of severe erosion under current conditions. This approach aims to establish a nuanced management framework that accounts for the unique vulnerabilities arising from land-use juxtaposition, offering a replicable strategy for similar complex watersheds globally.

## Materials and methods

### Description of the study area

The Qara-Su watershed, covering approximately 4,861 km², is located in the southern part of the Ardabil Plain and serves as one of the most important hydrological sub-watersheds feeding the Aras River in northwestern Iran. The watershed exhibits considerable natural diversity, with elevations ranging from about 1,200 m in the downstream areas to over 4,200 m in the highlands. Steep slopes in the eastern and western regions, along with an average overall slope of 16.5%, play a significant role in erosion dynamics and sediment production. The watershed’s drainage network includes three main rivers (Qara-Su, Ghuri-Chay, and Neor) each with numerous tributaries that influence the hydrological structure of the region. Part of the Fandoghlu Forest, one of the area’s valuable ecosystems, lies in the upper portions of the watershed and affects both vegetation patterns and hydrological behavior.

**Fig. 1:**
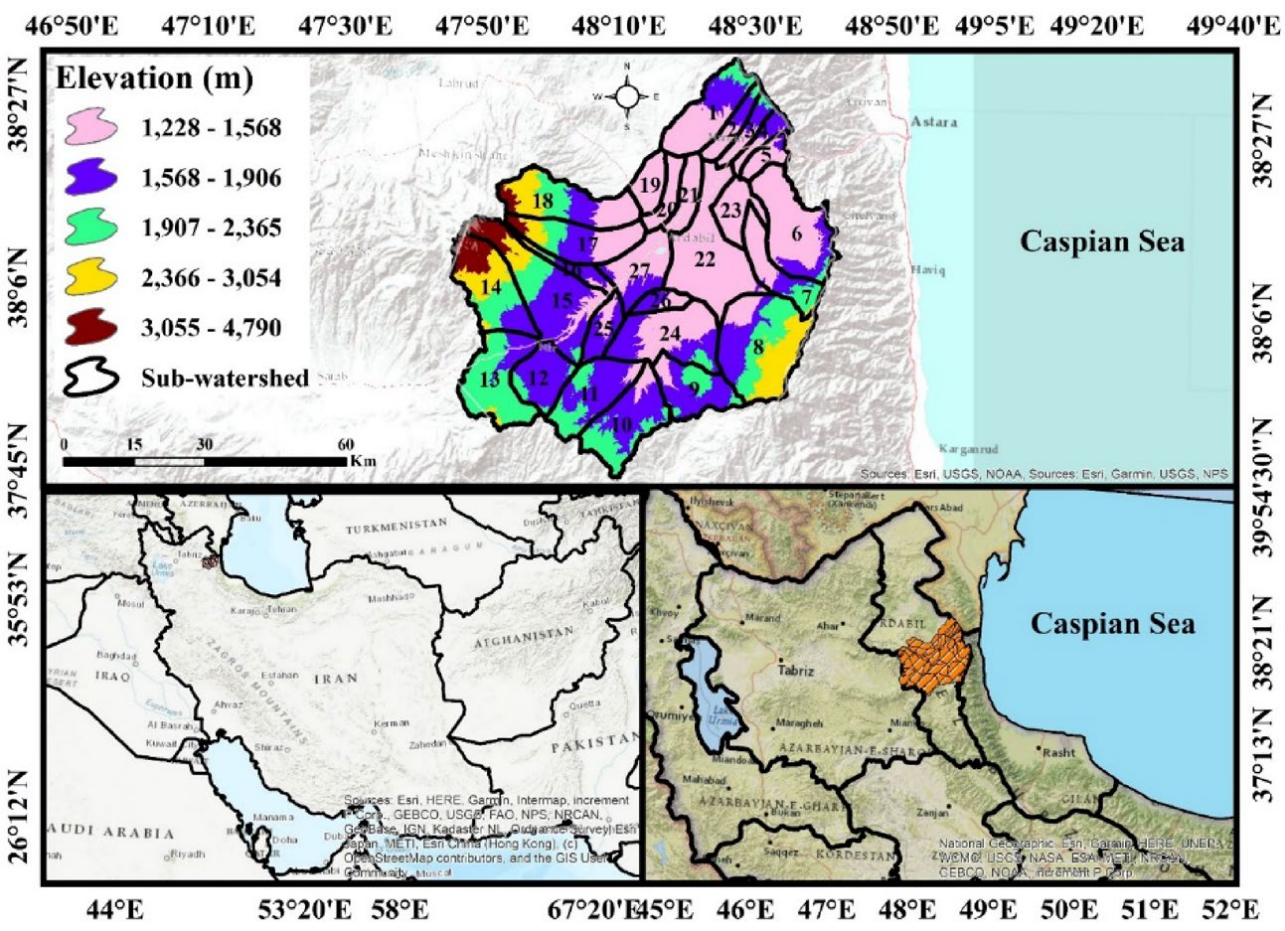
Geographic location of the Qara-Su watershed in Ardabil Province and northwestern Iran (Map processing and creation were carried out by the researchers using ArcMap within ArcGIS version 10.8^31^https://www.esri.com/en-us/arcgis/products/arcgis-desktop/overview).

Climatically, the Qara-Su watershed receives an average annual precipitation of about 351 mm, reflecting semi-arid to temperate conditions. This, combined with rugged topography, land-use patterns, and soil characteristics, indicates the importance of assessing surface erosion in the region. Geographically, the watershed is situated between 47°42’–48°28’ E longitude and 37°40’–38°20’ N latitude and encompasses extensive agricultural lands, rangelands, and mountainous areas in Ardabil Province^30^. The location of the Qara-Su watershed in Iran and Ardabil Province is shown in Fig. 1. The sub-watersheds were delineated using a standard GIS-based hydrological toolset. The process relied on a high-resolution Digital Elevation Model (DEM) to accurately derive flow direction, flow accumulation, and the stream network. The resulting watershed boundaries were defined by the natural topographic divides (ridge lines) and the connectivity of the drainage system.

### Research methodology

#### Soil erosion modeling

The RUSLE model (Revised Universal Soil Loss Equation) (Eq. 1) is one of the most widely used empirical models for estimating water erosion^32,33^. It is based on five main factors: rainfall erosivity (R), soil erodibility (K), slope length and steepness (LS), vegetation cover and management (C), and conservation practices (P). Using climate data, soil properties, topography, and land-cover patterns, the model calculates annual soil loss and is extensively applied in GIS and remote sensing environments. Despite its broad use, RUSLE is primarily designed for sheet and rill erosion and does not directly simulate more severe forms such as gully erosion^34,35^. The components of the RUSLE model are described below.

1 $${\mathrm{A}}\,{\mathrm{=}}\,{\mathrm{R}}{\text{. K}}{\text{. LS}}{\text{. C}}{\text{. P}}$$

where A is the amount of soil lost due to sheet and rill erosion (tons per hectare per year), R is rainfall erosivity (MJ·mm/ha·hr·year), K represents soil erodibility and the inherent susceptibility of the soil (t·h/MJ·mm), LS is the topographic factor (dimensionless), C is the vegetation cover factor (dimensionless), and P is the soil conservation practice factor (dimensionless)^36^.

##### Rainfall erosivity (R)

With climate change, rainfall intensity is expected to increase in many regions, potentially leading to higher soil erosion rates. Therefore, continuously updating R factor data using modern technologies such as remote sensing and artificial intelligence is essential for more accurate erosion modeling^37^. The rainfall erosivity factor (R) represents the energy and intensity of rainfall and its role in soil erosion. It is calculated based on the relationship between rainfall intensity and duration, indicating the capacity of rainfall to detach and transport soil particles^34^. Wischmeier’s method, due to its simplicity and applicability in diverse regions, is among the most widely used approaches for estimating this factor ^38^. The R factor was calculated using Eq. (2).

2 $$\:R=\sum\nolimits_{i=1}^{12}1.735\times\:{10}^{\left(1.5\times\:{\mathrm{log}}_{10}\left(\frac{{p}_{i}^{2}}{p}\right)\right)-0.8188}$$

Where (p_i_) represents the monthly precipitation and (p) the annual precipitation. In this study, monthly and annual precipitation data for a 40-year period (1981–2022) were used.

##### Soil erodibility (K)

Soil erodibility (K) reflects the inherent susceptibility of soil to water erosion and is influenced by factors such as soil texture, organic matter content, permeability, and structural properties. In this study, K was determined based on soil texture, land-use type, and the Curve Number (CN). Using CN as an indicator of soil hydrological condition and infiltration capacity allows for better classification of land units, as soils with higher CN generally have lower permeability and higher erodibility. Combining CN with land-use and soil texture provided an integrated framework for estimating K and enabled more accurate identification of spatial variations in soil erodibility across the watershed^39,40^. While classical methods for calculating K, such as the Wischmeier and Smith^41^ nomograph, as well as advanced approaches like the EPIC model and laboratory soil tests^42^, have been widely used, integrating CN and soil texture is particularly suitable for areas with high land heterogeneity. Table 1 presents the K values assigned for the study area.

**Table 1:** Assigned K values for the study area. [Adapted from^41^.

Soil Type	Hydrologic Group	Soil Erodibility (K)
Sandy Loam	B	0.25
Loam	C	0.30
Sandy Clay Loam	C	0.25
Clay	B	0.20

##### Topographic factor (LS)

The LS factor represents the combined effect of slope length (L) and slope steepness (S) on surface soil erosion. Increasing slope length and steepness accelerates runoff and enhances soil particle transport, leading to higher erosion rates. This factor is typically calculated using a Digital Elevation Model (DEM) and empirical-topographic relationships. Common methods include the equations of Moore & Burch^43^ and Desmet and Govers^44^, where slope length and steepness are derived from elevation data and computed continuously across the watershed^43^.

3 $$\:LS={\left(Flow\:\mathrm{A}\mathrm{c}\mathrm{c}\mathrm{u}\mathrm{m}\mathrm{u}\mathrm{l}\mathrm{a}\mathrm{t}\mathrm{i}\mathrm{o}\mathrm{n}\:\times\:\frac{Cell\:Size}{22.13}\right)}^{0.4}{\left(\frac{sin(Slope\times\:3.14/180)\times\:0.017450}{0.0896}\right)}^{1.3}$$

##### Land cover management (C) factor

The C factor represents the influence of vegetation cover, land-use type, and management practices on soil erosion. Dense vegetation, such as forests and healthy rangelands, minimizes the C factor by reducing raindrop impact energy and slowing runoff. In contrast, bare agricultural lands, fallow fields, and uncultivated areas typically exhibit the highest C values. Generally, data for calculating this factor are derived from satellite imagery and vegetation indices such as NDVI and SAVI^45^. In this study, the NDVI was extracted from Landsat 8 (OLI sensor) imagery acquired on 5 June 2024. Image processing and NDVI calculation were performed using TerrSet software to capture vegetation cover at peak growth season with the highest possible accuracy.

4 $$\:\mathrm{C}=\left[\frac{\left(-\mathrm{N}\mathrm{D}\mathrm{V}\mathrm{I}+1\right)}{2}\right]$$

##### Conservation practices (P) factor

The P factor represents the effect of soil conservation methods and practices in reducing runoff velocity and controlling erosion. These measures play a crucial role in preventing soil loss by regulating surface water flow and decreasing runoff energy^46^. The P factor ranges from 0.1 (highly effective practices) to 1 (no conservation measures). Traditionally, P values were assigned using standard tables, but in recent years, simulation models and GIS-based assessments have been employed for more accurate estimation^47^.

For land-use mapping of the study area on 5 June 2024, Landsat 8 (OLI/TIRS) satellite imagery was used, obtained from the USGS database. The raw images were pre-processed in ENVI software, including radiometric correction to remove sensor and atmospheric effects and geometric correction using ground control points (GCPs) for accurate alignment with base maps. Supervised classification was then applied to distinguish land-use types, with main classes including irrigated and rainfed agriculture, residential areas, rangelands, water bodies, and rock outcrops. Maximum Likelihood classification was employed in ENVI to generate the final raster land-use map, which was then imported into ArcGIS version 10.8 for post-processing, including noise removal, merging of similar classes, and boundary smoothing to improve spatial accuracy. Class accuracy was validated using Google Earth imagery, and overall accuracy and Kappa coefficient were used as standard metrics for reporting classification reliability^48^.

In this study, the P factor was determined by combining the land-use map and slope map (percent slope) in ArcGIS version 10.8. Each slope and land-use class was assigned a P value according to Table 2, and the final layer was converted to raster format. Specifically, P values for orchards and rangelands were set at 0.7 and 0.9, respectively.

**Table 2:** P values for different land-use types and slope classes^49^.

Land-use Type	Slope (%)	*P* Value
Water bodies	0–33	*P* = 0 as a mask
Irrigated agriculture	0–33	0.05
Rainfed agriculture	0–5	0.11
	5–10	0.12
	10–20	0.14
	20–30	0.19
	30–50	0.25
	> 50	0.33
Forest	0–33	0.08
Other land uses	0–33	1.00

#### Calculation of Moran’s I and hotspot analysis

To examine the spatial distribution of erosion values and identify significant spatial patterns, spatial autocorrelation indices were used. Moran’s I is one of the most widely recognized indices, ranging from + 1 to − 1. Values close to + 1 indicate positive clustering or high spatial similarity (e.g., High–High or Low–Low patterns), while values near − 1 indicate spatial dispersion and neighborhood dissimilarity^50^. Values near zero suggest a random spatial pattern^8,51^.

In this study, Global Moran’s I was used to assess the overall spatial distribution of erosion across the watershed, while Anselin Local Moran’s I identified areas with spatial behaviors differing from their neighbors. Local Moran’s I calculates Z-scores and significance levels (p-values) to reveal HH clusters (high values surrounded by high values) and LL clusters (low values surrounded by low values)^52^. Additionally, the Getis-Ord Gi* statistic was applied to detect areas of intense spatial concentration of high or low values. Hotspots are regions with high values surrounded by similarly high values, whereas coldspots are areas with low values adjacent to other low-value locations^53^.

## Results and discussion

### Results of soil erosion modeling

**Fig. 2:**
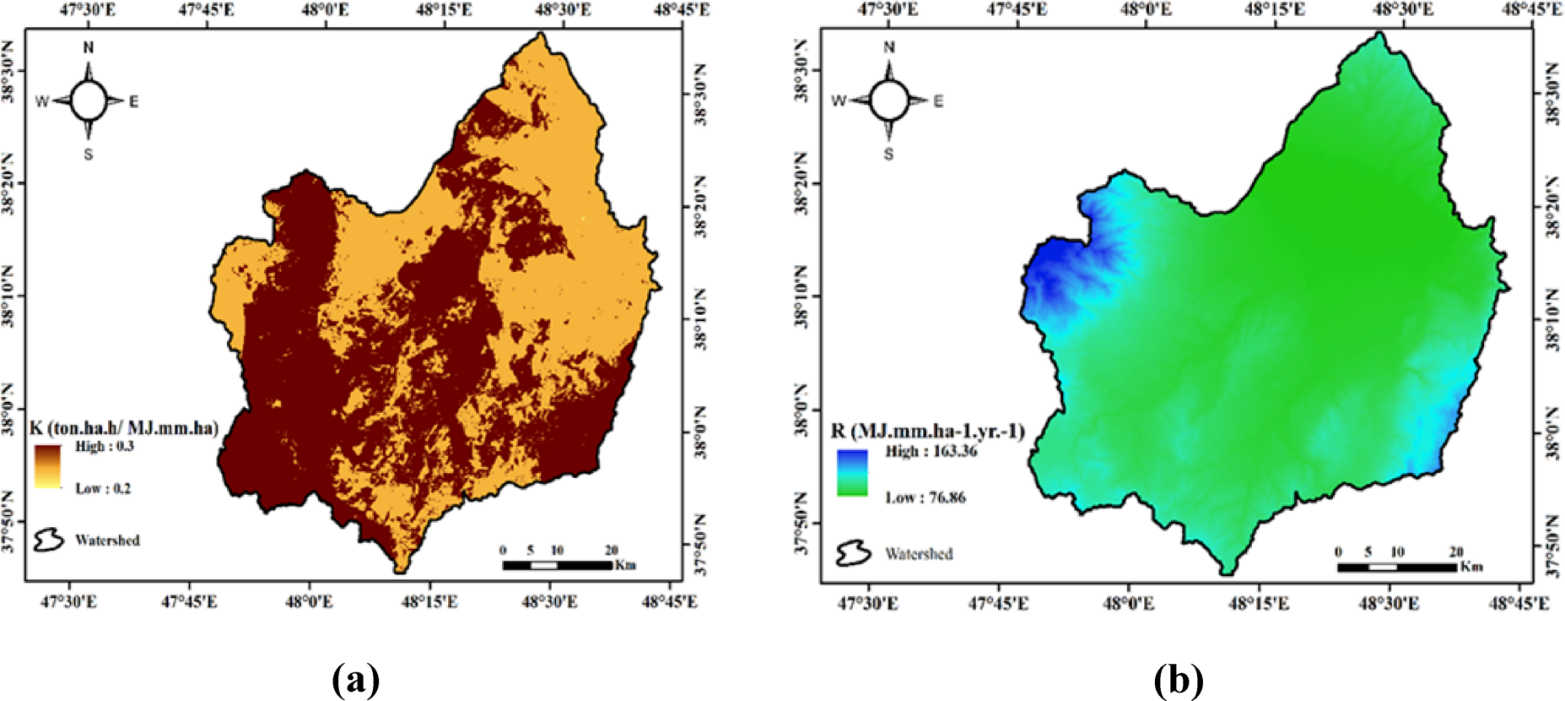
Spatial distribution of erosion-related factors in the Qara-Su watershed: **(a)** soil erodibility factor (K), **(b)** rainfall erosivity factor (R) (Map processing and creation were carried out by the researchers using ArcMap within ArcGIS version 10.8^31^https://www.esri.com/en-us/arcgis/products/arcgis-desktop/overview).

The results of the K and R parameters for estimating soil erosion in the Qara-Su watershed are presented in Fig. 2.

As shown in Fig. 2, the R factor in the studied watershed varies from 76.87 to 163.36 MJ·mm/ha·hr·year, with an average value of 78.08 MJ·mm/ha·hr·year. This range indicates a significant difference in the intensity and energy of rainfall across the watershed. Areas with higher R values are typically exposed to more intense rainfall systems, prolonged precipitation, and short-duration heavy rainfall events. Conversely, lower values are found in drier areas with milder rainfall. Spatial analysis showed that Sub-watershed 14, with an R value of 107.72 MJ·mm/ha·hr·year, has the highest sensitivity to erosive rainfall, possibly due to its exposure to weather fronts or specific elevations that enhance rainfall intensity. In contrast, Sub-watershed 21, with an R value of 78.57 MJ·mm/ha·hr·year, has the lowest value, likely due to its location in a region with weakened rainfall systems or reduced raindrop energy. This difference reflects the microclimatic influence on spatial variations in rainfall erosion across the watershed.

The soil erodibility factor (K) indicated that fine-textured and low-permeability soils have the highest sensitivity. This result aligns with results by Alewell et al^20^. who reported that sensitive soils in intensively farmed areas have higher K values. In the study area, higher K values were observed in regions dominated by agricultural land use or areas with degraded vegetation cover.

According to the results in Fig. 2, the K factor in the watershed ranges from 0.2 to 0.3 ton.ha.h/MJ.mm.ha, with an average value of 0.28 ton.ha.h/MJ.mm.ha. This range suggests that parts of the watershed have soils that are relatively sensitive to detachment and particle transport. Areas with high K values typically have finer soil textures (more silt and clay), unstable soil structures, and lower permeability, all of which increase the intrinsic erodibility of the soil. Sub-watershed 13 shows the highest K value of 0.30 ton.ha.h/MJ.mm.ha, indicating the presence of fine-textured soils, lower organic matter, and a more fragile structure. In contrast, Sub-watershed 5, with a K value of 0.25 ton.ha.h/MJ.mm.ha, demonstrates the lowest erodibility, likely due to the presence of coarser, more stable soils with a stronger structure. This variation in K values shows the heterogeneous soil texture composition in the watershed, which leads to differences in erosion patterns.

**Fig. 3:**
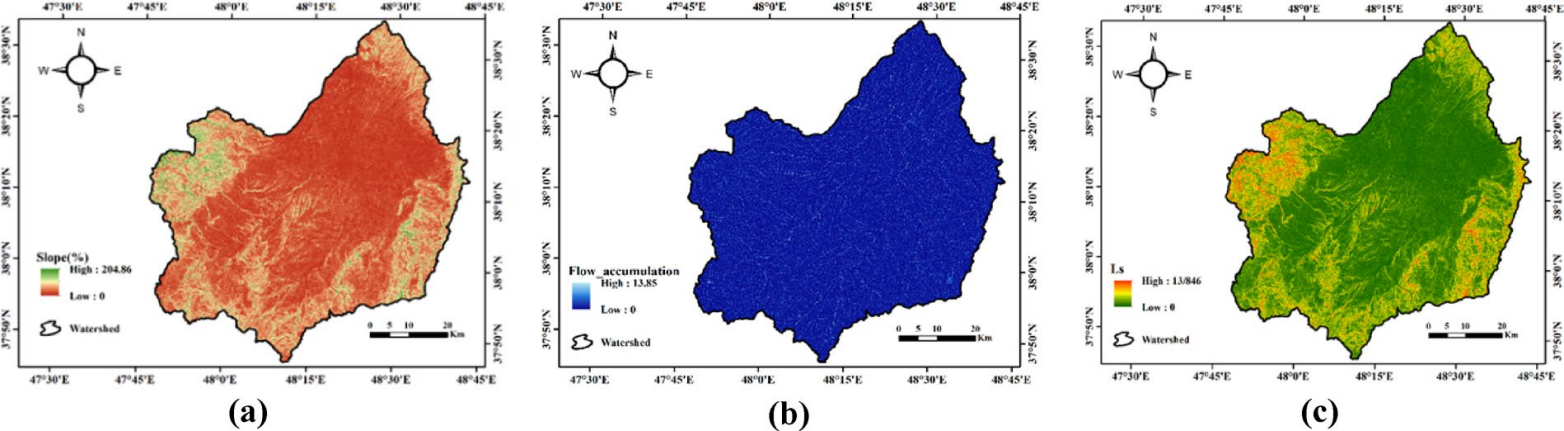
Spatial distribution of topographic factors in the Qara-Su watershed: **(a)** slope, **(b)** flow accumulation, **(c)** LS factor (Map processing and creation were carried out by the researchers using ArcMap within ArcGIS version 10.8^31^https://www.esri.com/en-us/arcgis/products/arcgis-desktop/overview).

Maps of slope, flow accumulation, and the LS factor are presented in Fig. 3.

The slope map (in percentage) of the studied watershed (Fig. 3) shows spatial distribution of slopes over the study area. This indicates significant topographic relief, which considerably increases the LS factor in the RUSLE model. According to the results in Fig. 3, the topographic factor LS in the studied watershed ranges from 0 to 13.84, with an average value of 1.62. This indicates a considerable variation in slope and slope length conditions across the watershed. High LS values are mainly observed in areas with steep slopes, long slopes, and a considerable distance from watercourses. These conditions increase runoff speed and sediment transport capacity, thereby intensifying erosion. Conversely, the lowest LS values are found in flat areas and regions with gentle slopes, where surface flow is slower and the potential for soil particle detachment and movement is lower. Sub-watershed analysis revealed that Sub-watershed 14, with a value of 3.04, has the highest LS, indicating higher susceptibility to erosion based on topographic conditions. In contrast, Sub-watershed 21, with a value of 0.54, shows the lowest LS, suggesting more stability and gentler slopes in this area.

**Fig. 4:**
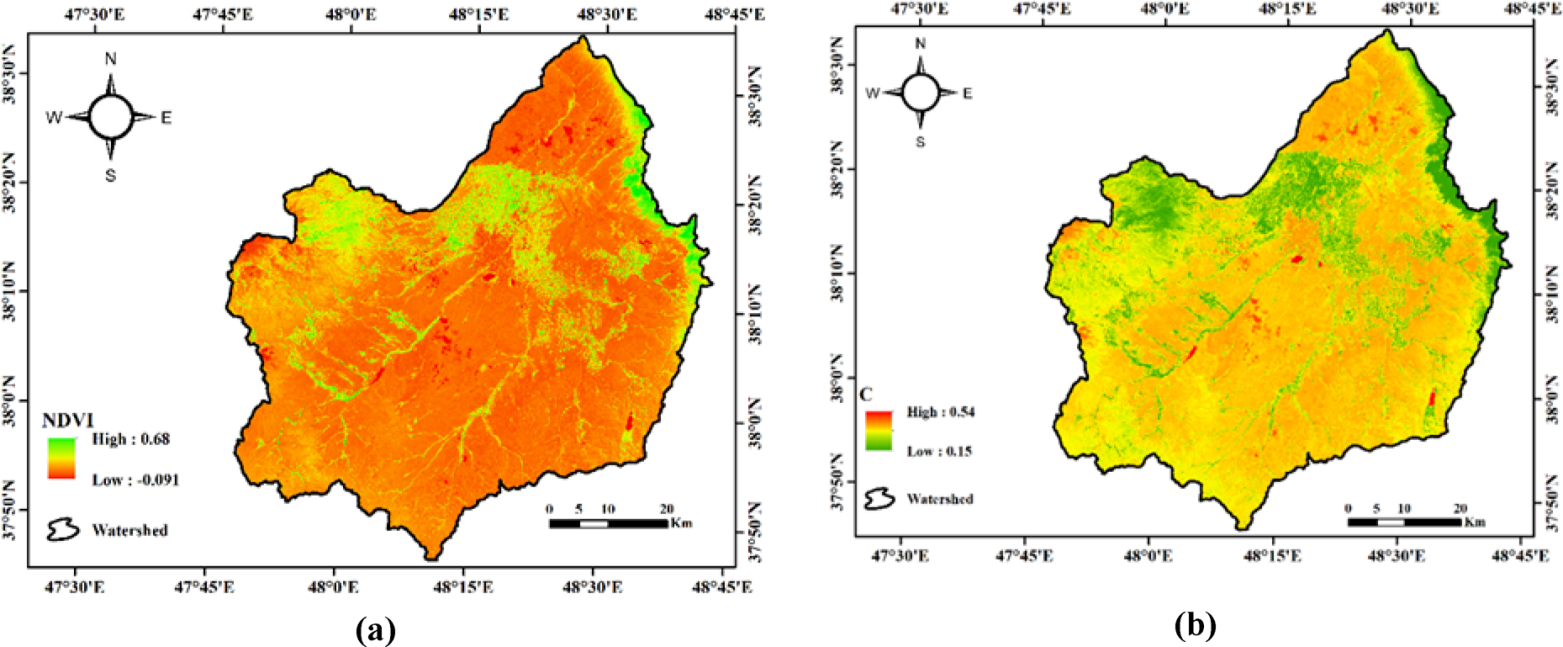
Spatial distribution of vegetation-related factors in the Qara-Su watershed: **(a)** NDVI index, **(b)** C factor (Map processing and creation were carried out by the researchers using ArcMap within ArcGIS version 10.8^31^https://www.esri.com/en-us/arcgis/products/arcgis-desktop/overview).

The results of calculating the C parameter and the normalized vegetation cover difference index in the Qara-Su watershed are presented in Fig. 4.

The map of the normalized difference vegetation index (NDVI) for the Qara-Su watershed (Fig. 4) shows the spatial distribution of vegetation ranging from 0.09 (lowest, areas without cover in the south and east) to 0.69 (highest, dense areas in the north). This indicates a high ecological diversity, from forests and well-covered rangelands to barren lands prone to erosion. This pattern, with weak coverage concentrated on the steep slopes, indicates the high potential for soil erosion (linked to the C factor in the RUSLE model). According to the results (Fig. 4), the land cover management and vegetation factor C in the studied watershed ranges from 0.16 to 0.55, with an average value of 0.39. This variation shows that vegetation cover and land-use types differ significantly across the watershed. Areas with weak vegetation cover, barren lands, or agricultural areas without vegetation show the highest values for C, as in these areas, the energy of raindrop impact is directly transferred to the soil surface, increasing the detachment and transport of soil particles. In contrast, the lowest values of C are observed in areas with dense vegetation, rangelands, or orchards, where the protective cover reduces runoff initiation and speed and protects the soil surface. Sub-watershed analysis further revealed that Sub-watershed 25, with a value of 0.41, has the highest C value and thus higher vulnerability in terms of vegetation cover, whereas Sub-watershed 20, with a value of 0.35, shows the lowest value, indicating better cover and its effective role in reducing erosion. The analysis of the C factor showed that vegetation cover plays a decisive role in controlling erosion, with areas without cover or with low NDVI values having the highest C values. This result aligns with studies by Xo et al^23^. and various reports in Iran, including Bayat^16^ and Ahmadabadi^17^.

**Fig. 5:**
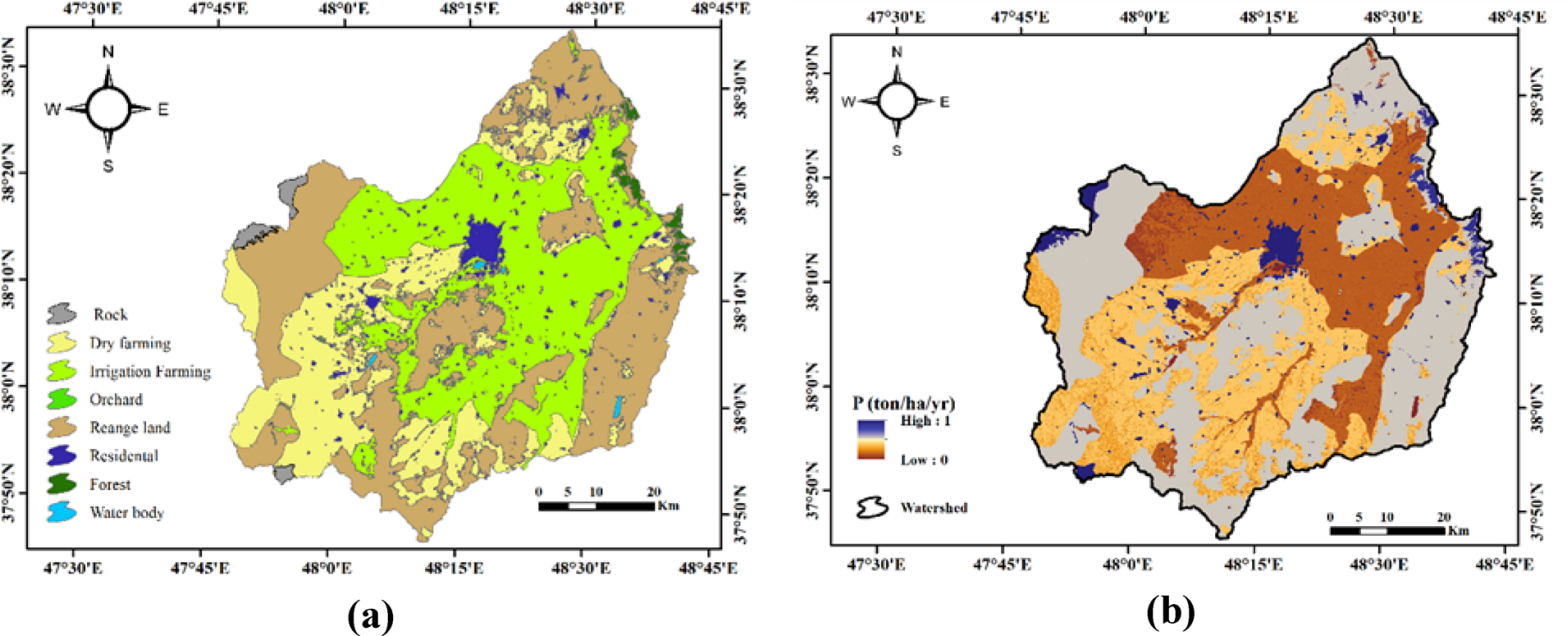
Spatial distribution of management and land-use factors in the Qara-Su watershed: **(a)** land use, **(b)** P factor (Map processing and creation were carried out by the researchers using ArcMap within ArcGIS version 10.8^31^https://www.esri.com/en-us/arcgis/products/arcgis-desktop/overview).

The results of the P parameter and land use map in the Qara-Su watershed are shown in Fig. 5.

Based on the results shown in Fig. 5, the soil conservation factor (P) in the studied watershed varies between 0 and 1, with an average value of 0.36 tons/hectare/year. This range indicates that the intensity and extent of conservation practices implemented in different parts of the watershed vary significantly. A P value close to zero indicates the presence of effective soil conservation methods such as terracing, contour plowing, strip cropping, and other management practices, which reduce runoff velocity and consequently significantly decrease erosion. In contrast, a P value close to 1 indicates a lack of conservation measures, with these areas being more vulnerable to water erosion. Sub-watershed analysis revealed that Sub-watershed 3, with a value of 0.48 tons/hectare/year, has the highest P value, indicating the least conservation management, while Sub-watershed 19, with a value of 0.15 tons/hectare/year, has the lowest P value, showing better conservation performance. This spatial distribution suggests that implementing conservation measures in certain sub-watersheds can play a key role in reducing erosion and controlling sediment, and can help prioritize management actions for the watershed. Similar results were observed in the studies of Hailu et al. (2023) and Munye et al. (2024), which demonstrated that management actions and the integration of spatial analyses enhance the accuracy of identifying high-risk areas. According to the P map, sub-watersheds lacking conservation management were classified as high-erosion zones, which also overlapped with the hotspots at the 95% and 99% confidence levels. This alignment suggests that conservation measures can directly reduce erosion intensity in critical areas.

The land use map achieved a Kappa coefficient of 82% and an overall accuracy of 89%. According to the land use classification, the areas of orchard, water body, rocky outcrop, forest, irrigated agriculture, rainfed agriculture, and rangeland, as well as residential areas, account for 3.56, 9.01, 39.87, 39.12, 1425.43, 1022.19, 1569.30, and 127.90 square kilometers, respectively, in the Qara-Su watershed.

### Spatial soil erosion mapping

**Fig. 6:**
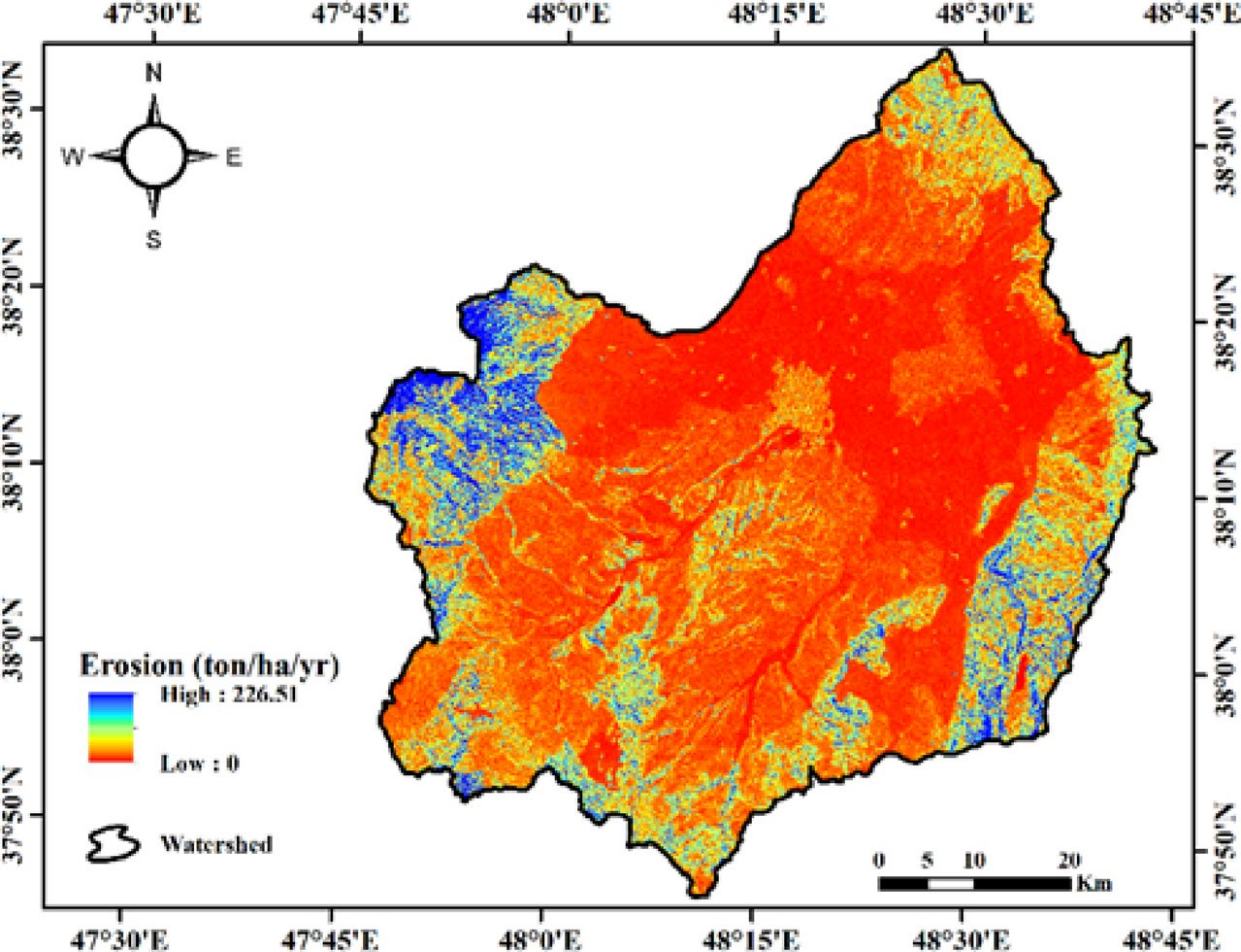
Results of erosion estimation using the RUSLE model in the Qara-Su watershed (Map processing and creation were carried out by the researchers using ArcMap within ArcGIS version 10.8^31^https://www.esri.com/en-us/arcgis/products/arcgis-desktop/overview).

The final map of annual erosion, derived from the RUSLE model for soil erosion estimation, is shown in Fig. 6.

The final map of annual erosion derived from the RUSLE model, shown in Fig. 6, reveals that the erosion rate in the studied watershed ranges from 0 to 226.51 (this is a local maximum, not the average) tons per hectare per year. The spatial erosion pattern indicates that a significant portion of the watershed area falls within moderate to very high erosion classes. Areas with high erosion are primarily located on the northern, northwestern slopes, and parts of the central highlands; these regions have steep slopes, long slope lengths, and are dominated by rainfed agriculture, which increases the LS and C factors, thereby exacerbating erosion. In contrast, areas with low to very low erosion are mostly found in relatively flat land, foothill areas, and parts of the western regions, characterized by gentle slopes, more stable soils, and adequate vegetation cover. This pattern shows that the spatial distribution of erosion is directly related to the combination of LS, C, and K factors, with the highest vulnerability observed in areas lacking vegetation cover and those with long slopes. Overall, the results indicate that erosion in significant areas of the watershed is at concerning levels and requires the implementation of management and conservation measures. Areas with very high erosion can be identified as critical zones, and watershed management and vegetation restoration efforts should prioritize these regions.

**Fig. 7:**
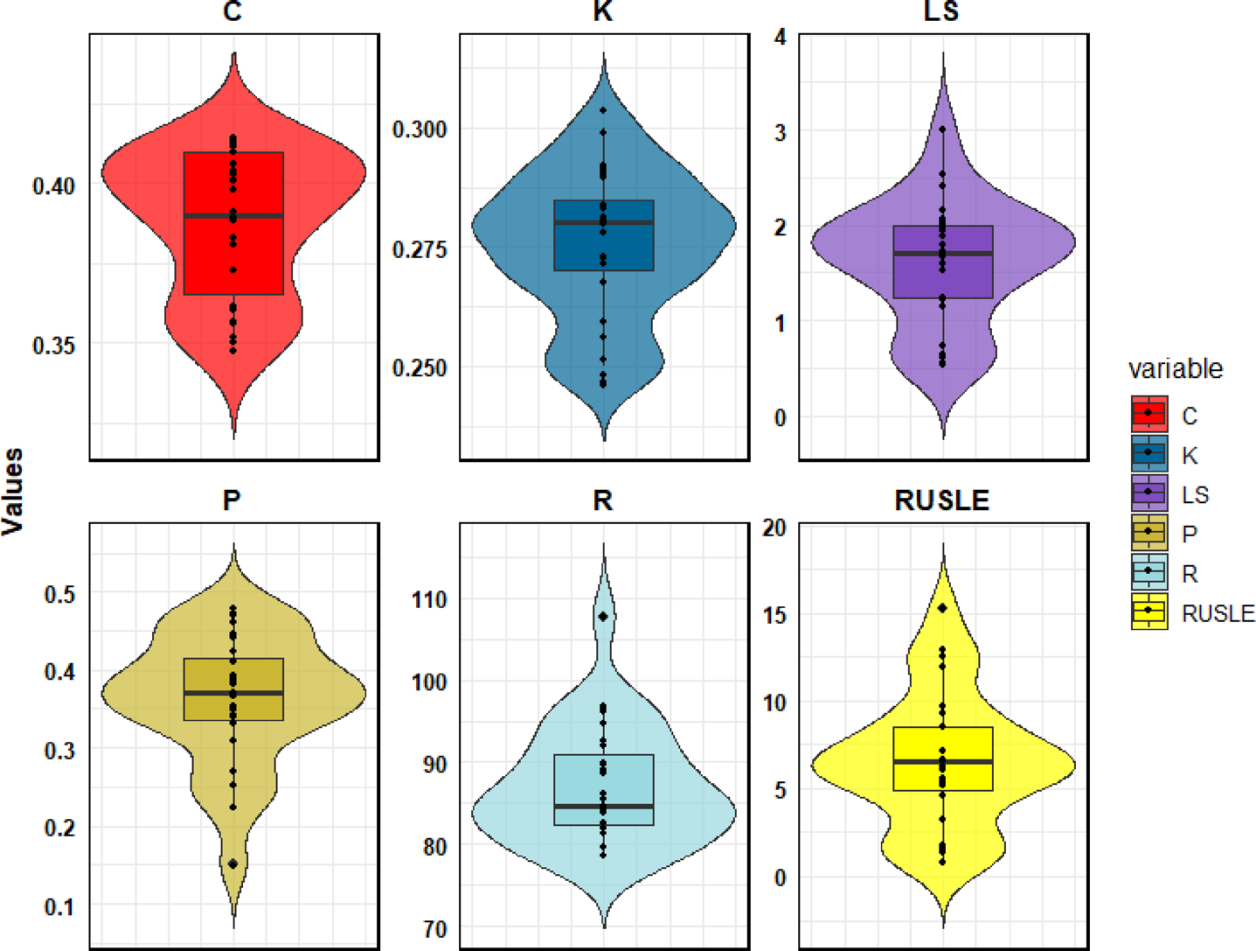
Average values of RUSLE factors (R, K, LS, C, P) across the sub-watersheds of the Qara-Su watershed, R-factor: (MJ·mm/ha·hr·year), K-factor: (t·h/MJ·mm), LS-factor, C-factor, and P-factor are Dimensionless.

The distribution of average RUSLE model parameters (R, K, LS, C, and P) across the sub-watersheds of the Qara-Su watershed is presented in Fig. 7.

### Interpretation RUSLE factors in erosion patterns across sub-watersheds

The analysis of the average RUSLE factor values across the sub-watersheds (Fig. 7) reveals a clear relationship between the combination of these factors and the final erosion rates (Fig. 6). Sub-watersheds with the highest erosion rates (such as sub-watersheds 14 and 15) generally are characterized by the simultaneous combination of high values in several factors. For instance, sub-watershed 14, which is a 99% confidence hotspot, has very high values for the topographic factor (LS = 3.04) and rainfall erosivity (*R* = 107.7 MJ·mm/ha·hr·year), coupled with a relatively weak vegetation cover factor (C = 0.38). This combination intensifies runoff energy and reduces surface soil resistance, ultimately leading to a very high erosion rate (average of 15.2 tons per hectare per year). In such high-erosion sub-watersheds, the topographic factor (LS) plays a dominant and determining role. Areas characterized by long and steep slopes have become severe erosion hotspots primarily due to high runoff energy, even with only moderate C and K values.

In contrast, sub-watersheds with the lowest erosion rates (such as the cold spot cluster 19–23) typically exhibit low values of key factors. Specifically, these sub-watersheds benefit from gentle slopes (low LS values), less erosive rainfall (low R values), and better vegetation cover or more effective conservation management (low C and P values). For example, sub-watershed 21 has the lowest LS value (0.54) and one of the lowest R values (78.6), which, alongside relatively suitable vegetation cover, results in the lowest erosion rate among the mountainous sub-watersheds. Here, the stabilizing effect of gentle topography and better vegetation cover dissipates erosive energy effectively.

This analysis confirms that in a watershed with complex topography and land use like Qara-Su, no single factor alone controls the erosion pattern; rather, it is the interaction and synergistic effect of multiple factors that shapes the critical hotspots. However, the dominance of the LS factor is the key to interpreting the high sensitivity of the western and southwestern sub-watersheds. This understanding enhances the practical value of the study by providing a quantitative basis for targeted management. It indicates that control measures must be prioritized according to the dominant factor combination in each sub-watershed, for instance, prioritizing runoff control and mechanical operations (e.g., terracing) on high-LS lands over purely biological interventions (e.g., increasing vegetation cover) in areas where topography is the primary driver.

### Spatial autocorrelation (Moran’s I) and hotspot analysis

**Fig. 8:**
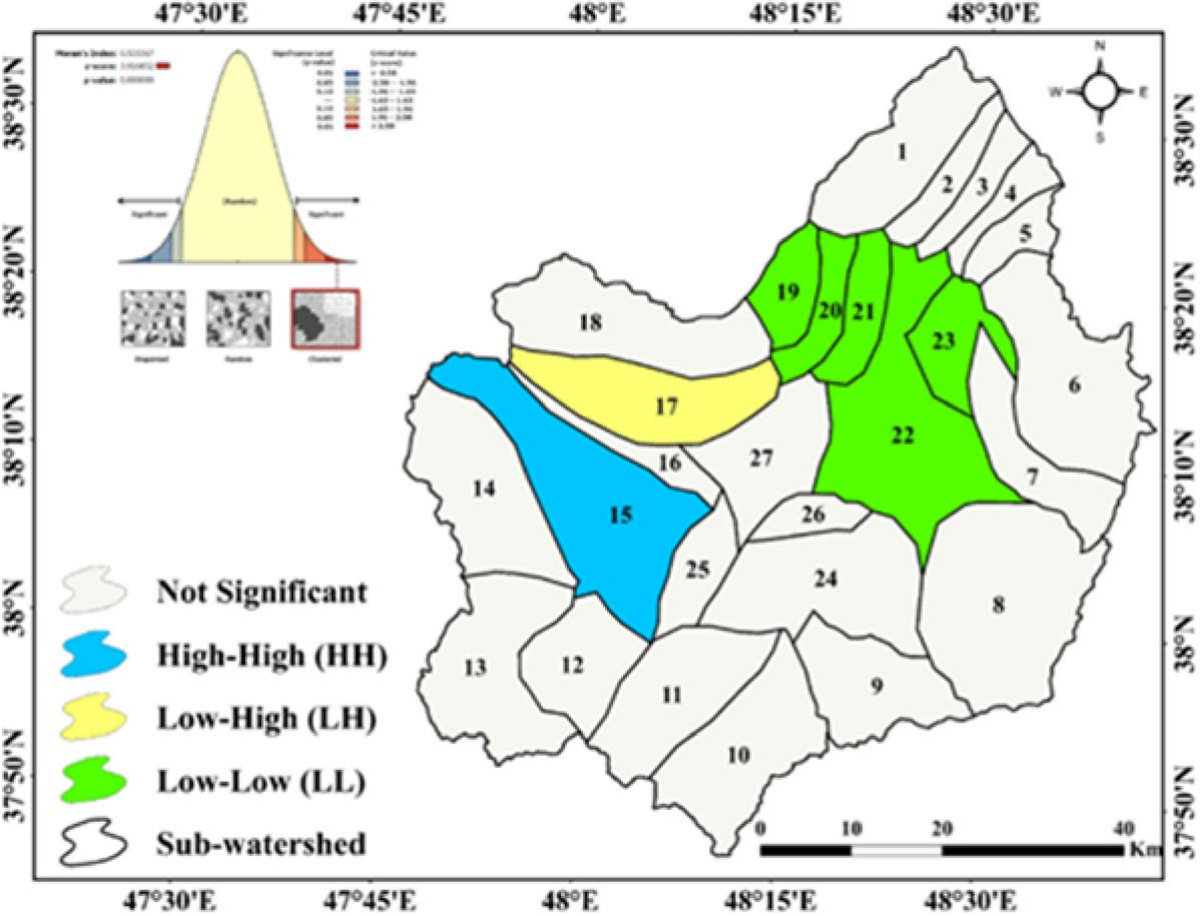
Moran’s I analysis in the sub-watersheds of the Qara-Su watershed (Map processing and creation were carried out by the researchers using ArcMap within ArcGIS version 10.8^31^https://www.esri.com/en-us/arcgis/products/arcgis-desktop/overview).

**Fig. 9:**
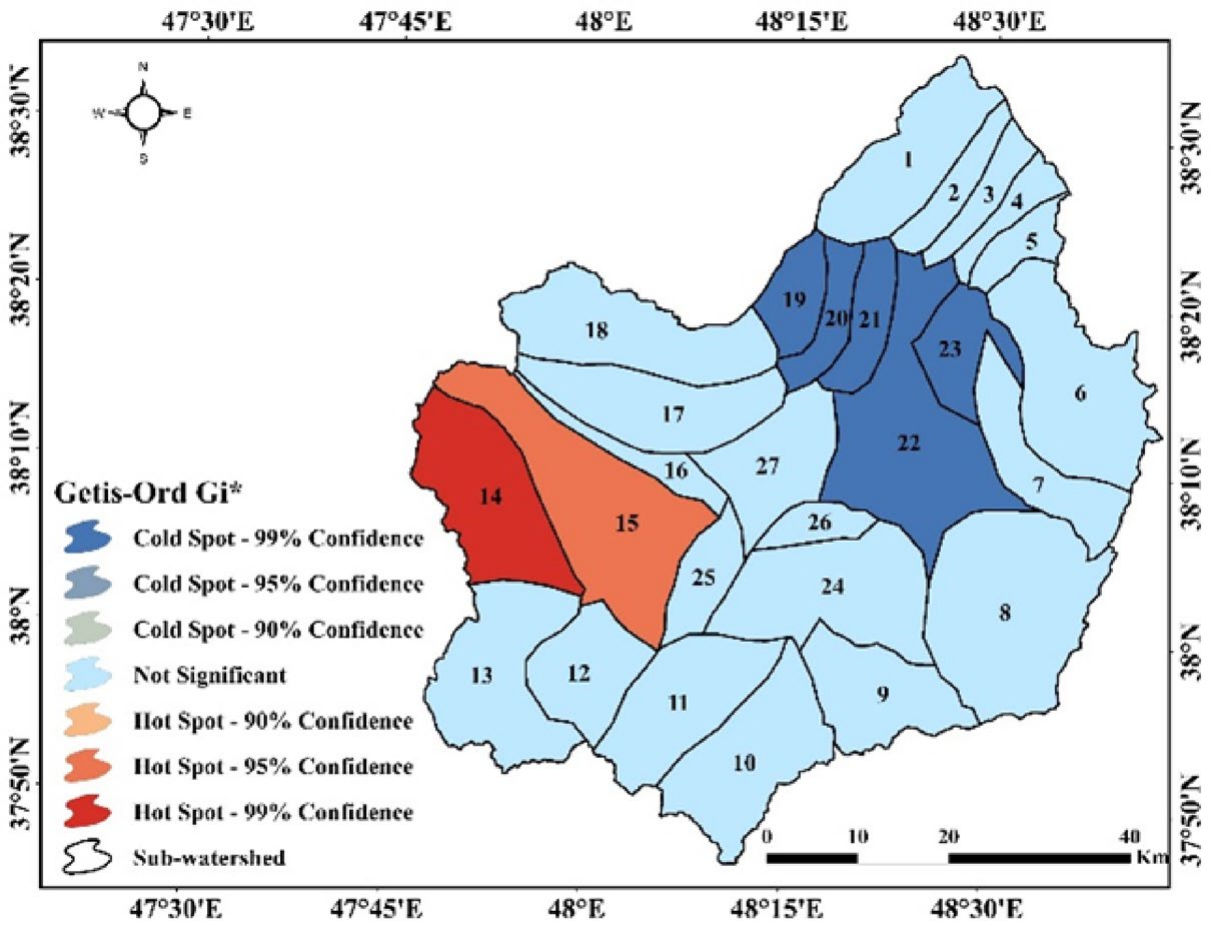
Hotspot and coldspot analysis in the sub-watersheds of the Qara-Su watershed (Map processing and creation were carried out by the researchers using ArcMap within ArcGIS version 10.8^31^https://www.esri.com/en-us/arcgis/products/arcgis-desktop/overview).

The results of Moran’s I spatial autocorrelation and hotspot/coldspot analysis for the sub-watersheds of the Qara-Su watershed are presented in Figs. 8 and 9.

The Moran’s I and Getis-Ord Gi* analyses objectively identified significant clusters of high erosion (hotspots) in the southwestern part of the basin (sub-watersheds 14 and 15). A quantitative assessment of the average RUSLE factor values in these critical sub-watersheds provides direct evidence: The average rainfall erosivity factor (R) in sub-watershed 14 (identified as a hotspot with 99% confidence) was 107.72 MJ·mm/ha·hr·year, which is more than 37% higher than the basin-wide average (78.08). Concurrently, the average topographic factor (LS) in this same sub-watershed (3.04) is nearly double the basin-wide average (1.62). In contrast, the average values for the C and K factors in these critical sub-watersheds do not show a similar deviation from the overall mean (e.g., C in sub-watershed 14 is 0.38 compared to the average of 0.39). This clear spatial correlation between high-erosion clusters and the quantitatively higher values of R and LS (compared to the other factors) serves as direct quantitative evidence that strengthens the initial qualitative conclusion.

Local Moran’s I (Anselin Local Moran’s I) results revealed significant clustering of soil erosion within the watershed, indicating a non-random spatial pattern. Sub-watersheds 19, 20, 21, 22, and 23 were classified as LL clusters, reflecting concentrations of low erosion values and high stability. In contrast, sub-watershed 15 was identified as an HH cluster, representing extremely high erosion concentrations and one of the watershed’s most critical areas. Sub-watershed 17 fell into the LH category, suggesting that although its current erosion is low, it is adjacent to high-erosion areas and may be prone to future changes.

The Getis-Ord Gi* hotspot analysis confirmed these patterns. Sub-watersheds 19–23 were identified as coldspots with 99% confidence, representing the most stable areas, whereas sub-watershed 15 was classified as a 95% confidence hotspot and sub-watershed 14 as a 99% confidence hotspot, indicating regions of concentrated erosion and their critical status. The overlap between Moran’s I clusters and hotspot results indicates that the western and southwestern parts of the watershed have the highest sediment production potential and erosion intensity, while the northeastern and eastern regions remain relatively stable.

The results of the spatial analyses (Local Moran’s I and Getis-Ord Gi*), explicitly reporting the statistical confidence levels, enable evidence-based prioritization. Accordingly, significant high-erosion clusters (hotspots) were identified in sub-watersheds 14 and 15 with confidence levels of 99% (p-value ≤ 0.01) and 95% (p-value ≤ 0.05), respectively. Conversely, stability clusters (coldspots) were confirmed in sub-watersheds 19–23 with a 99% confidence level. These confidence levels quantify the probability that the identified patterns are not random, providing a robust scientific basis for a hierarchical management framework. Within this framework, immediate priority (Tier 1) is assigned to hotspot sub-watersheds with 99% confidence (e.g., sub-watershed 14), requiring rapid implementation of intensive control measures (such as bio-mechanical operations and vegetation restoration). High priority (Tier 2) includes hotspots with 95% confidence (e.g., sub-watershed 15), necessitating targeted interventions. Stable coldspot areas with high confidence are designated as reference or protected areas (Tier 3), primarily requiring monitoring and preventative management. This approach enables the optimal allocation of limited management resources to the most critical areas, thereby maximizing the overall efficiency of erosion control strategies at the basin scale.

The location of HH clusters and hotspots on steep, sparsely vegetated, and unstable land aligns with results from similar studies, such as Tamene et al^19^. in Ethiopia and Xo et al^23^. in China, which reported that areas with steep slopes, vegetation loss, and land-use changes have the highest likelihood of forming erosion hotspots. This spatial pattern analysis, based on current erosion rates, identifies statistically significant clusters of severe erosion. Therefore, these areas (e.g., sub-watersheds 14 and 15) should be prioritized for immediate conservation interventions.

### Limitations

The assessment of soil erosion in this study is based on the RUSLE model, which effectively estimates sheet and rill erosion for spatial comparison. However, the results represent average annual soil detachment potential, not actual sediment delivery to the watershed outlet, as RUSLE does not account for sediment transport, deposition, or channel processes. It also excludes discrete events like gully erosion and landslides, and may underrepresent extreme storms due to its use of long-term average rainfall data. These limitations clarify the study’s scope (prioritizing sub-watersheds by erosion susceptibility) and highlight needed future work. Integrating sediment delivery models, high-resolution climate data, and gully erosion assessments would enable a more complete sediment budget.

## Conclusion

The results of this study demonstrated that the RUSLE model, by simultaneously incorporating climatic, physiographic, soil, and management factors, effectively estimates for spatially estimating water erosion and identifying critical zones at the watershed scale. Analysis of the contributing factors showed that rainfall erosivity (R) and topographic factor (LS) have the greatest influence on erosion intensity and spatial distribution, with areas experiencing heavy rainfall, steep slopes, and long slope lengths exhibiting the highest erosion values. The soil erodibility factor (K) indicated that fine-textured, low-permeability, and weak soils are more susceptible to erosion, whereas more stable soils recorded lower erodibility. Vegetation cover (C) revealed that the absence or weakness of vegetation directly increases erosion, while dense cover and protected land uses play a key role in erosion reduction. The conservation practice factor (P) confirmed that implementing measures such as terracing, contour farming, and other soil-protective practices can significantly reduce erosion rates. Spatial analyses using Local Moran’s I and the Getis-Ord Gi* hotspot model clarified the erosion patterns. Moran’s analysis revealed significant clustering, identifying HH clusters in the southwest as highly critical zones and LL clusters in the north, northeast, and east as stable, low erosion rate areas. Hotspot analysis confirmed this pattern, designating the southwest as a high-confidence hotspot and the east and northeast as coldspots with low erosion intensity. These results show the highly heterogeneous nature of erosion distribution, shaped by the interplay of topography, rainfall, vegetation cover, and land management. Overall, the variations among sub-watersheds demonstrate that erosion results from the combined effect of multiple factors, not a single driver. Consequently, effective watershed management should prioritize sub-watersheds characterized by high R and LS values, sensitive soils, and weak vegetation cover. Implementing conservation measures (such as vegetation restoration, slope stabilization, land-use control, and targeted planning) can substantially reduce erosion damage. Furthermore, integrating erosion modeling with spatial analyses offers a powerful tool for sustainable watershed management.

Finally, the results of this study provide a foundation for more sophisticated analytical pathways in future research. To move beyond the parametric limitations of empirical models like RUSLE and to better decipher the complex, non-linear interactions among erosion drivers, the adoption of machine learning and artificial intelligence approaches is highly recommended. Recent studies have successfully demonstrated the superior predictive power of machine learning algorithms in modeling spatial soil erosion and identifying dominant controlling factors at various scales^54–56^. Future work should focus on developing integrated ML frameworks that can process high-dimensional geospatial data, including high-resolution topographic indices, time-series remote sensing data for vegetation and moisture, climate projections, and detailed land-use change layers. Such data-driven models are particularly promising for dynamic erosion hotspot forecasting, quantifying the individual and interactive contributions of numerous natural and anthropogenic factors, and ultimately supporting adaptive, predictive watershed management strategies in the face of climate and land-use change.

## Data Availability

All data generated or analyzed during this study are included in this published article.
